# Apoptotic MSCs and MSC-Derived Apoptotic Bodies as New Therapeutic Tools

**DOI:** 10.3390/cimb44110351

**Published:** 2022-10-24

**Authors:** Irina V. Kholodenko, Roman V. Kholodenko, Alexander G. Majouga, Konstantin N. Yarygin

**Affiliations:** 1 Laboratory of Cell Biology, Orekhovich Institute of Biomedical Chemistry, 119121 Moscow, Russia; 2 Laboratory of Molecular Immunology, Shemyakin–Ovchinnikov Institute of Bioorganic Chemistry of the Russian Academy of Sciences, 117997 Moscow, Russia; 3 Faculty of Chemical and Pharmaceutical Technologies and Biomedical Products, Mendeleev University of Chemical Technology of Russia, 125047 Moscow, Russia

**Keywords:** mesenchymal stem/stromal cells, apoptosis, apoptotic bodies, programmed cell death, MSC-based therapy, extracellular vesicles, immunomodulation

## Abstract

Over the past two decades, mesenchymal stem cells (MSCs) have shown promising therapeutic effects both in preclinical studies (in animal models of a wide range of diseases) and in clinical trials. However, the efficacy of MSC-based therapy is not always predictable. Moreover, despite the large number of studies, the mechanisms underlying the regenerative potential of MSCs are not fully elucidated. Recently, it has been reliably established that transplanted MSCs can undergo rapid apoptosis and clearance from the recipient’s body, still exhibiting therapeutic effects, especially those associated with their immunosuppressive/immunomodulating properties. The mechanisms underlying these effects can be mediated by the efferocytosis of apoptotic MSCs by host phagocytic cells. In this concise review, we briefly describe three types of MSC-generated extracellular vesicles, through which their therapeutic functions can potentially be carried out; we focused on reviewing recent data on apoptotic MSCs and MSC-derived apoptotic bodies (MSC-ApoBDs), their functions, and the mechanisms of their therapeutic effects.

## 1. Introduction

The therapeutic potential of mesenchymal stem cells (MSCs) was discovered decades ago. However, despite extensive preclinical and clinical studies during such a long period of time, the molecular and cellular mechanisms underlying the beneficial effects of MSC transplantation have not been fully elucidated. Initially, it was assumed that transplanted MSCs exert therapeutic activity mainly through homing into damaged tissues [1,2] with subsequent differentiation into specialized types of targeted tissue cells (site-specific differentiation) replacing the dead or damaged cells [3,4,5]. Later, this mechanism of action was questioned since the site-specific differentiation of transplanted MSCs in the recipient’s tissues was shown to be an exceptionally rare event [6]. At the next stage of research targeted at the mechanisms of MSC action, it was found that after homing to the injury site, the transplanted MSCs are able to recruit resident stem/progenitor cells, which ensure subsequent tissue regeneration [7,8]. This recruiting ability was associated with the paracrine effects of MSCs, which produce a large number of trophic factors including cytokines, chemokines, and growth factors. In addition to resident progenitor cells recruiting to the damaged area, the paracrine effects of MSCs were also associated with their immunomodulatory abilities, the suppression of apoptosis of the resident cells in injured tissues, stimulation of angiogenesis, and enhancement of other regeneration-related processes [9,10]. By this time, some studies have reported a lack of the migration of transplanted cells and their rapid death [11,12]. Based on these discoveries, it was suggested that the transplantation of MSCs themselves is not necessary for the implementation of their regenerative potential, and, perhaps, conditioned media from MSCs could exhibit similar effects. Indeed, a number of studies have shown that MSC-conditioned media really enhance regeneration and mimic other MSC effects in various disease models [13,14,15]. Consequently, a new direction of biomedical research concentrated on the use of MSC-derived exosomes as therapeutic agents started to develop [16]. At the same time, data reliably demonstrating that transplanted MSCs undergo rapid clearance in the body of the recipient animals and do not have the ability for any long-term and effective homing have been steadily accumulating [11,17]. It was also found that apoptotic MSCs exhibit therapeutic effects, in particular, a pronounced immunomodulation activity [18]. Currently, an increasing number of researchers are inclined to believe that the apoptosis of transplanted MSCs is necessary for the manifestation of their regenerative and therapeutic potential. Accordingly, the administration of MSC-ApoBDs instead of whole live cells is being tested. However, apoptosis of MSCs after transplantation is still generally considered a disadvantage or as an event that negatively affects therapeutic efficacy, and researchers seek to increase the survival of transplanted cells in vivo [19,20,21]. Probably, the two opposite approaches can be reconciled based on the accumulating results of preclinical and clinical studies demonstrating that live MSCs and apoptotic MSCs are not therapeutically interchangeable, but rather are effective in different contexts and probably through different mechanisms [22].

This concise review presents essential data regarding the types of extracellular vesicles (EVs) produced by MSCs, including exosomes, microvesicles, and apoptotic bodies (MSC-ApoBDs). Their main characteristics, biogenesis, and biomarkers are briefly described, and issues related to the differential separation of various types of EVs are considered. The focus of this review is on MSC apoptosis and clearance after transplantation, the biodistribution of apoptotic MSCs, and the therapeutic potential of MSC-ApoBDs. The results of the works presented in the review suggest the following hypothetic mechanism of the therapeutic action of MSCs: (1) the transplanted cells, undergoing apoptosis, release large amounts of ApoBDs; (2) ApoBDs and/or apoptotic MSCs are predominantly engulfed by resident phagocytes and exert certain physiological effects locally; (3) a certain amount of ApoBDs and/or apoptotic MSCs is able to enter the circulation and spread to other organs and tissues, where they are also subsequently phagocytosed by various cell types, causing changes in their phenotype and/or stimulating their activation, which ultimately leads to the manifestation of therapeutic and regenerative effects.

## 2. MSC-derived Extracellular Vesicles: Exosomes, Microvesicles, and Apoptotic Bodies

There are three main types of EVs, namely exosomes, microvesicles, and apoptotic bodies. They all are some kind of containers, surrounded by a lipid bilayer and containing a whole range of biologically active molecules, including RNA, DNA, proteins, and lipids. EVs participate in intercellular communication, regulation of tissue homeostasis, immunomodulation, and other physiological processes. Different types of EVs differ in their biogenesis, size, content, surface markers, and mechanisms of action. The main characteristics of the three types of EVs are presented below.

### 2.1. Exosomes

Exosomes are nanosized EVs that are produced by all types of living cells in the body [23]. Exosomes are found in body fluids, where their content and composition can change significantly with the development of various pathophysiological conditions. The study of the composition and content of exosomes in biological fluids can serve as a diagnostic and/or predictive biomarker of various pathologies, including oncological [24,25], viral [26,27], autoimmune [28], and neurodegenerative [29,30] diseases.

The biogenesis of exosomes has been described in detail in several reviews [31,32]. Briefly, exosomes are formed within the living cell by inward budding of the late endosomal membrane, resulting in the formation of multivesicular bodies. After that, the membrane of multivesicular bodies fuses with the plasma membrane of the cell, thus releasing exosomes into the extracellular space.

Exosomes are the smallest type of EVs, their sizes vary from 20 to 200 nm in diameter, regardless of the type of cells that they originate from [33,34]. Exosomes have established marker proteins, the presence of which is characteristic of almost all exosomal vesicles originating from various cell types and biological fluids. These markers include tetraspanins, CD9, CD63, and CD81, as well as annexin II and V, flotillin-1 and -2, heat shock proteins 70 and 90, TSG101, and others [35]. The exosome contents are largely determined by the type of cells producing them, the state of differentiation of these cells, and the cellular microenvironment. Haraszti et al. [36] showed that the exosome proteome patterns of two cell lines of human glioblastoma U87 and human hepatocarcinoma Huh7 are significantly similar while differing remarkably from the exosome proteome of human MSCs. At the same time, the Huh7- and MSC-derived exosome lipidomes turned out to be largely similar, but very different from the U87-derived exosome lipidome [36]. In addition to proteins and lipids, exosomes serve as containers for the transfer of a whole range of small RNAs, such as miRNAs and tRNAs. Baglio et al. [37] demonstrated that exosomes of the adipose-derived MSCs (AD-MSCs) predominantly carry tRNA halves and are practically devoid of full-length transcripts, while exosomes of the bone marrow-derived MSCs (BM-MSCs) include two different tRNA length profiles. Exosomes generated by BM-MSCs that appear to carry high levels of the key stemness markers (Sox2, POU5F1A/B, and Nanog) contain both full-length transcripts and 33-bp tRNA fragments, while more differentiated cells show the same tRNA length profile as the AD-MSC exosomes [37]. Under simulated ischemic tissue conditions, MSCs produce elevated exosome numbers and these exosomes contain more angiogenic signaling proteins than those derived from MSCs under standard culture conditions [38]. The functions of exosomes in the physiological and pathological processes, as well as the potential for their therapeutic and diagnostic use, are described in detail in several reviews [39,40,41].

### 2.2. Microvesicles

Unlike exosomes, microvesicles are formed by direct outward budding of the cell plasma membrane and subsequent pinching of the vesicles into the extracellular space. In a sense, they can be considered “physiological liposomes” [42]. The diameter of microvesicles is about 100–1000 nm, larger than the diameter of exosomes [43]. The microvesicle formation and shedding are the results of a complex process of cytoskeleton reorganization and the loss of the membrane bilayer physiological asymmetry, which leads to the exposure of phosphatidylserine (PS) to the outer membrane leaflet of microvesicles [44]. Since microvesicles are buds of the plasma membrane, they contain the membrane components of the cells from which they originated, as well as their cytoplasmic contents [45]. For example, MSC-derived microvesicles exhibit on their surface such MSC markers as CD90, CD44, and CD73 [46], while microvesicles derived from endothelial progenitor cells expose CD133 and CD271 [47]. Based on the mechanism of microvesicle biogenesis, it was suggested that they should contain no or very small amounts of proteins specifically associated with organelles, including the Golgi apparatus, mitochondria, endoplasmic reticulum, and nucleus [48]. However, recent proteomic studies showed that microvesicles are enriched in the endoplasmic reticulum, proteasome, and mitochondrial proteins [36]. Similar to exosomes, microvesicles expose tetraspanins CD9 and CD63 on their surface [49]. The functions and therapeutic potential of MSC-derived microvesicles have been thoroughly analyzed [50,51,52].

### 2.3. Apoptotic Bodies

Unlike exosomes and microvesicles, the ApoBDs are generated not by normal live cells, but by cells undergoing programmed cell death. Most ApoBDs have diameters of 0.5–5 μm; significantly larger than exosomes and microvesicles [53,54]. However, during apoptosis smaller (50–500 nm) exosome-like EVs are also released [55], though their biogenesis is still not fully elucidated [56]. Thus, the full range of ApoBDs sizes covers two orders of magnitude—50–5000 nm [57].

#### 2.3.1. ApoBD Biogenesis

Apoptosis is the main mechanism of programmed cell death in normal and cancer cells [58,59]. Cells that die by apoptosis go through several morphological stages: plasma membrane blebbing and cell shrinkage, nuclear fragmentation, condensation, and fragmentation of genetic material (chromatin and nucleosomal DNA), and breakdown of cellular contents into separate membrane-enclosed vesicles called ApoBDs [58]. At the early stage of apoptosis, cells undergo a characteristic contraction, the size of the cells becomes smaller, and the contents inside the cells become more densely packed. Simultaneously, another phenomenon known as pycnosis (irreversible condensation of chromatin) occurs. The early stage of apoptosis is followed by karyorrhexis, the fragmentation of the nucleus. Karyorrhexis is followed by further plasma membrane blebbing, resulting in the disintegration of the apoptotic cells into the ApoBDs. Ihara et al. [60] proposed a scheme illustrating the process of ultrastructural change leading to the formation of ApoBDs. At stage I, “crescent-shaped spaces” form around the nucleus; chromatin condenses unevenly. At stage II, the nuclei are moderately invaginated, then vesicles appear. At stage III, the nuclear buds pinch, forming fragments, and gradually condense. The cytoplasm also condenses and shrinks. At stage IV, the chromatin of the fragments is compacted. Then the ApoBDs are formed. Thus, ApoBDs contain remnants of apoptotic cells, i.e., cytoplasm, organelles, and nuclear contents. The components of apoptotic cells are randomly distributed between the ApoBDs, and this distinguishes them from the other types of EVs (exosomes and microvesicles), where the distribution of biologically active molecules occurs by regulated coordinated cellular mechanisms [61]. The packaging of intracellular organelles, such as mitochondria, into apoptotic bodies, is a stochastic process [62,63]. Therefore, specific organelles or nuclear contents may or may not be present in a particular ApoBD. The cargo of each individual ApoBD consists of cellular components that have ended up in a cytoplasmic protrusion that created this particular ApoBD. Therefore, some ApoBDs are almost completely loaded with condensed nuclear chromatin, while others carry only cytoplasmic components, including intact organelles [58]. Such an irregular distribution of cellular content between ApoBDs leads to the variability of their physical properties. For example, tightly packaged DNA is denser compared with plasma membrane-enclosed cytoplasm and carries a negative charge. Depending on the cargo, ApoBDs carry a wide variety of biomolecules. Based on their contents, ApoBDs are classified into two groups: if they carry fragments of the apoptotic nucleus, they are called nuclear or DNA-bearing ApoBDs, and if they carry cytoplasm, they are called cytoplasmic ApoBDs [64].

One of the earliest and most morphologically identifiable stages of the formation of ApoBDs is membrane blebbing. Blebs are formed due to increased hydrostatic pressure inside the cell after contraction mediated by the actin-myosin cytoskeleton [65]. Blebbing is a dynamic process that consists in continuously repeating multiple protrusions and retractions of the apoptotic cell plasma membrane [66]. Thus, two events occurring at the early stage of apoptosis are responsible for the formation of blebs: protrusion and retraction, leading to cell shrinkage, known as apoptotic volume decrease [67]. Caspases play a key role in these events, as well as in the whole process of apoptotic cell death. Cell contraction and blebbing are caused by forces generated by the actin-myosin cytoskeleton, activated by the phosphorylation of myosin light chains by caspase-cleaved Rho-associated coiled-coil kinase (ROCK1) [68,69]. Caspase-mediated cleavage and weakening of the structural nuclear protein laminin [70,71], as well as the contraction generated by the actin-myosin cytoskeleton, lead to nuclear fragmentation and chromatin redistribution into blebs and subsequently into ApoBDs [66]. More details about the mechanisms of membrane blebbing are described by Julian and Olson [72].

However, for many cell types, it has been shown that ApoBDs are formed not due solely to blebs, but involve other forms of membrane protrusions. In T cells and thymocytes, Poon et al. [73] found the ‘string-like’ membrane protrusions (known as apoptopodia) that are formed exclusively after membrane blebbing and facilitate the separation of blebs to generate individual ApoBDs. These authors later described a new mechanism of ApoBD generation from monocytes via the formation of a “beads-on-a-string” membrane structure (named “beaded apoptopodia”) [74]. Structures similar to beaded apoptopodia have also been found in osteosarcoma cells after cell death induction [75]. On a wide range of human cell lines, Moss et al. [76] showed that in addition to actomyosin, microtubules play an important role in the formation of ApoBDs. This process has been studied in detail in the A431 cell line where the induction of apoptosis leads to the formation of small cellular protrusions or spikes, which eventually grew by more than 20 µm. During fragmentation ApoBDs often remained attached to the spikes, and, as a result, apoptotic cells took on very irregular profiles [76]. Unfortunately, at present, the mechanism of ApoBD formation by MSCs that have undergone programmed cell death remains unclear.

#### 2.3.2. ApoBDs Clearance

In most cases, the engulfing of apoptotic cells and their remnants is referred to as phagocytosis, although the more correct term is “efferocytosis” to distinguish it from complement-mediated phagocytosis. Apoptotic cells and ApoBDs secrete and/or concentrate on their surface two types of signals recognized by the phagocytic cells. The first type of signals, the so-called “find me” signals, include secretion/expression by apoptotic cells/ApoBDs of such factors as ATP and UTP nucleotides [77], chemokine CX3CL1 [78], lysophosphatidylcholine [79], sphingosine 1-phosphate [80], and phosphatidylserine-bound (PS-bound) endogenous chemokines [81]. The “find me” signals serve as attractants for phagocytes including monocytes, macrophages, and dendritic cells, enabling the prompt clearance of the dying cells [82]. The second type of signals are called the “eat me” signals. They mark apoptotic cells and cellular debris as objects that should be cleared. The clearance is mediated by specific interactions between recognition receptors on phagocytes and specific changes in the composition of apoptotic cell membranes [83,84,85]. Among these changes, the most characterized involves the translocation of PS from the inner to the outer leaflet of the lipid bilayer during the apoptotic process [86]. The translocated PS can be directly recognized by phagocytes via specific receptors, for example, Tim4 in mice [87] and CD300 in humans [88,89]. In addition, PS on the surface of ApoBDs can interact with certain molecules, which, in turn, bind to the corresponding receptors on phagocytic cells, thus forming bridges between ApoBDs and phagocytes. Serum proteins such as growth arrest-specific 6 (Gas6) protein and protein S (PROS1), specifically bind PS and TAM receptors (Tyro3, Axl, and MerTK) to form bridges between apoptotic cells and macrophages [86]. Annexin V can also serve as a bridge between PS on apoptotic cells and phagocytes [90]. Another well-characterized change in apoptotic cell membranes involves the oxidation of surface molecules. These changes create sites for the binding of thrombospondin [91,92] or complement protein C3b [93]. Thrombospondin and C3b are in turn recognized by the phagocyte receptors [94,95,96]. Such interactions between phagocytes and ApoBDs/apoptotic cells lead to the formation of a “phagocytic synapse” or “efferocytic synapse” [97]. Thus, efferocytosis marks the terminal stage of the apoptotic cycle and it is believed that the essence of this step is to prevent the development of secondary necrosis at the sites of massive apoptosis, the leakage of dangerous materials packed inside apoptotic cells into the environment and, as a consequence, to prevent inflammation and the development of autoimmunity.

### 2.4. Differential Detection of Diverse Types of EVs

Despite the increased interest of researchers in the study of EVs and, as a result, the exponential growth of the number of publications on this topic, specific markers that would distinguish or separate one type of EV from another have not yet been identified. In this regard, separation methods such as ultracentrifugation and density gradients, i.e., sorting EVs by size and density are still the most commonly used methods. Moreover, researchers have begun to use size exclusion chromatography and techniques based on tangential flow and microfluidics for EV separation [98]. However, these EV isolation methods do not allow to obtain pure fractions of a particular type of vesicles. As a rule, researchers isolate a mixture of different types of EVs with a relative enrichment of one of the vesicle types. This causes difficulties associated with the interpretation of the results, comparison of data obtained by different scientific teams, correct assessment of the physiological role of the three EV types, and their suitability for the development of therapeutic and diagnostic procedures. There is also certain confusion in terminology. Currently “EVs” is a collective term covering various subtypes of membrane structures released by cells and called exosomes, microvesicles, microparticles, ectosomes, oncosomes, apoptotic bodies, and many other names.

In 2014, the International Society for Extracellular Vesicles (ISEV) board members, based on their own experience, published an article detailing their recommendations on minimum experimental requirements for the determination of extracellular vesicles and their functions [99]. A list of minimum information for EV research (MISEV or MISEV2014) covering EV separation/isolation, characterization, and functional studies was provided. The main purpose of these recommendations was to draw the attention of researchers, as well as editors and reviewers, to the experimentation and reporting requirements that are specific to the EV field. When a significant amount of new scientific knowledge accumulates, the manuals are updated. For example, in 2018, an update of the document provided proposals for protein markers to confirm the presence of EVs. Recommendations on the EV nomenclature were also given. Thus, if authors are unable to identify specific markers of subcellular origin, it is recommended to consider using operational terms for EV subtypes that refer to physical characteristics, such as size or biochemical composition, or to resort to the description of isolation conditions or cells of origin instead of using terms such as exosome and microvesicle. Recommendations for determining the protein composition of EVs and evaluation of the functional properties have been updated and expanded [100].

The currently available research results regarding the characteristics of different types of EVs leave many unsolved problems. One of the main issues is the differential separation of vesicle subtypes in order to obtain pure, maximally homogeneous fractions. Identification of markers specific for each individual subtype of EVs could help in resolving this issue. Unfortunately, it is difficult to do. For example, it was initially assumed that tetraspanins are specific markers of exosomes, but later it was shown that these proteins are also present on microvesicles and ApoBDs [101]. Another example is the translocation of PS to the outer leaflet of the plasma membrane, which until recently was considered exclusively a sign of apoptosis, and PS as a marker of ApoBDs. It is now known that the same mechanism of rearrangement of the plasma membrane is a common feature during the formation of microvesicles [44,102]. PS exposure to the outer monolayer has also been shown in exosomes produced by tumor cells [103], oligodendrocytes [104], and T cells [105]. Thrombospondin could presumably serve as a marker of ApoBDs since it is an “eat me” signal [91,95], but recent studies have shown its exposure on exosomes [106,107]. Moreover, proteins directly involved in the progression of the apoptotic signal, for example, caspase 3, could serve as specific markers of ApoBDs [108,109]. However, the release of caspase 3 from cells as a cargo of EVs was also shown in the absence of apoptosis. This phenomenon is considered to be a mechanism aimed at protecting cells from apoptosis by ridding them of caspase accumulation [110,111].

In recent work, Zhang et al. [112] compared the proteomic profiles of ApoBDs and exosomes derived from three different types of MSCs, including human BM-MSCs, human AD-MSCs, and mouse BM-MSCs and showed that ApoBD protein patterns are 80% matched between BM-MSCs and AD-MSCs. More functional proteins were present in ApoBDs compared to exosomes obtained from the same cells. The authors found 13 proteins that were present in ApoBDs but not in exosomes, including Fas, Integrin alpha-5, Syntaxin-4, CD44, RhoA, Caveolin-1, Cavin1, Rab-5C, RPS25, Lamin B1, VDAC-2, Calnexin, and Calreticulin, as well as one protein present exclusively in exosomes, but not in ApoBDs, namely syntenin-1 [112]. The main characteristics of different types of EVs are summarized in Table 1.

These results demonstrate that the search for differential markers should include comparative studies of protein, as well as lipid patterns in different types of EVs isolated from the same cells in order to identify inclusion and exclusion biomarkers that will help to effectively separate pure fractions of individual EV subtypes. So far, the differential separation and characterization of various subtypes of EVs is based on the totality of many features, including isolation methods, size, density, protein, and lipid composition.

## 3. Methods of the MSC-Derived ApoBDs Isolation

Perhaps the main thing to remember with regard to the methods of ApoBDs isolation should be their advent from the cells that have undergone programmed cell death. Consequently, the ApoBD yield can be significantly increased by targeted induction of cell death, at least in the in vitro experiments. However, as mentioned above, apoptotic cells can also generate exosome-like EVs [55,56]; therefore, obtaining pure fractions of ApoBDs is a non-trivial task.

The preparation of ApoBDs from MSCs usually includes the induction of apoptosis with staurosporine doses of 250 nM–0.5 μM for 12 h, followed by centrifugation at 800–1000 g to remove cell debris and then at 16000 g to precipitate the ApoBDs [108,109,113]. 200 μM H_2_O_2_ for 8 h or nutrient deprivation by culturing MSCs in PBS for 24 h are also used as inducers of cell death in MSCs [112]. Le et al. [114] suggested using high hydrostatic pressure at 50 MPa for 36 h to induce MSC apoptosis ex vivo. The advantage of this method is that the cells are not exposed to any chemical reagents, while the efficiency of induction of apoptosis and the formation of ApoBDs is comparable to the induction of apoptosis in MSCs by 0.5 µM staurosporine [114]. Skovronova et al. [115] treated MSCs with 500 ng/mL anti-Fas Ab for 24 h to induce apoptosis. However, this method cannot always be used since it was shown that MSCs are resistant to Fas-induced apoptosis in vitro [116,117]. The described methods of the induction of apoptosis allow effective generation of ApoBDs varying in size from 100 nm to 1 μm in different studies. These ApoBDs show expression of C1q, Annexin V, PS, mesenchymal markers, including CD29, CD44, CD90, CD49e, CD146, and CD105, and EV markers CD9, CD63, and CD81, as well as the expression of cleaved caspase 3 [108,113,115].

## 4. Therapeutic Potential of Apoptotic MSCs or MSC-Derived ApoBDs

### 4.1. Biodistribution of Transplanted MSCs Undergoing Apoptosis In Vivo

As mentioned above, in the last few years, more and more works have appeared where it has been reliably proven that transplanted MSCs, regardless of the method of their administration, are quickly eliminated, while the therapeutic effects are preserved. These results led to the idea that transplanted cells do not need to remain viable for a long time in order to realize their therapeutic effects. Moreover, probably not live, but apoptotic cells are responsible for at least the immunomodulatory effects of MSCs transplantation.

As a rule, the bulk of the transplanted human MSCs isolated from bone marrow, adipose tissue, or the umbilical cord was found in the lungs of recipient animals within a few minutes after intravenous (i.v.) transplantation. Later, at 2 h after i.v. injection tiny numbers (less than 0.1%) of human MSCs were found in the liver. Transplanted human MSCs showed signs of apoptosis, including a decrease in cell size, caspase 3/7 expression, positive staining with annexin V, and co-expression of calreticulin, which is an “eat me” signal for phagocytic cells, as early as 30–60 min after the intravenous injection [118,119,120]. The number of living human MSCs progressively decreased in animals over time. All authors agree that human MSCs were completely eliminated and were not detected in the organs of recipient animals 24 h after transplantation [118,119,120]. Importantly, transplanted human MSCs that have undergone apoptotic cell death in animal bodies, i.e., in a xenogeneic environment, were co-stained with antibodies against the macrophage marker F4/80, the complement component C3b, the granulocyte marker GR-1, and the endothelial and platelet marker CD31/PECAM-1. These results indicate an interaction between human MSCs and different subpopulations of host phagocytic cells, which can potentially provide their rapid clearance [118].

As shown in several studies, the complete clearance of intravenously transplanted syngeneic MSCs was somewhat slower than in xenogeneic transplantation. The number of cells remaining alive decreased markedly after 12–24 h [121], and live cells were not detected after 7 days [122]. Similar results were obtained with intrapancreatic and intrasplenic transplantation of syngeneic MSCs when the complete clearance of transplanted cells occurred within 7 days. Moreover, transplanted cells were found in the liver 12 h after intrasplenic infusion, followed by a fall after 72 h [123]. In all the works described above, a significant increase in the expression of caspase 3 was noted in transplanted cells [121,122,123]. Subcutaneously injected syngeneic MSCs were detected a little longer (up to 7 days) with complete clearance after 14 days [122].

In the rat model of the brain injury, it was shown that intra-arterial infused human placenta MSCs [124] or human BM-MSCs [125] were not detected in rat brains 72 h after infusion. Transplanted cells were localized in the lumen of the blood vessels being in close contact with the vascular wall at 24–48 h after administration. At 72 h after transplantation, MSCs were phagocytosed, presumably, by activated microglia and macrophages, as shown by co-staining with CD44 and ED1 [125].

Thus, it has been shown that transplanted MSCs rapidly undergo apoptotic cell death followed by clearance due to host phagocytic cells, regardless of the route of administration, the source of MSCs, and even the genetic compatibility of the transplanted cells and the recipient animals. This raises several very important questions. First, what are the mechanisms of apoptosis induction in transplanted MSCs, and what factors trigger the process? Second, what phagocytic cells are able to ‘efferocytize’ apoptotic MSCs, and what are the consequences of this engulfing?

### 4.2. Apoptosis of Transplanted MSCs In Vivo

At present, very little is known about the mechanisms and molecular signals involved in triggering the cell death of transplanted MSCs. An important result of research in this area is the fact that apoptosis of transplanted MSCs is not the result of allogeneic and/or xenogeneic cell recognition since efficient elimination of infused cells occurs in syngeneic models as well. Galleu et al. [126] demonstrated that MSC apoptosis after transplantation was associated with the presence of effector cytotoxic CD8^+^Vβ8.3^+^ cells in the lungs of the GvHD recipient mice. Activated cytotoxic CD56^+^ NK cells and CD8^+^ T cells were found to be responsible for initiating MSC apoptosis. The molecular inducers of apoptosis included granzyme B and perforin and, to a lesser extent, FasL, while TRAIL was not involved in triggering the process [126]. The involvement of FasL/Fas in the induction of apoptosis of implanted MSCs was shown in a model of myocardial ischemia [19]. On the other hand, Pang et al. [120] tracked the presence of luciferase-expressing human MSCs in immunocompetent BALB/c mice and the two immunodeficient mouse models, NOD/SCID/Il2rγc^−/−^ (NSG) and BALB/c NOD.sirpa Rag2^−/−^Il2rγc^−/−^ (BRGS). The authors concluded that the rapid clearance of MSCs in the absence of the adaptive immune response and cytotoxic cells suggests that MSCs die in the lungs due to factors unrelated to immune cell function (for example, in this case, cell death was most likely triggered by nutrient deprivation/growth factors), and clearance occurs by myeloid cells without regard for the state of inflammation. Preda et al. [122] suggested that apoptosis of transplanted MSCs may be associated with microenvironmental conditions, including hypoxia and pro-inflammatory cytokines such as TNFα and IFNγ. In earlier studies, it was shown that human BM-MSCs, despite the expression of three cell-surface complement regulators (CD46, CD55, and CD59), activated the complement system and, as a result, were injured by membrane attack complexes, which led to the lysis of transplanted cells after transplantation into immunocompetent mice [127,128].

Based on the available data, it is still difficult to identify molecular factors inducing the apoptotic death of transplanted MSCs. Apoptotic stimuli likely depend on the localization of the infused cells (lungs, liver, brain, other organs), on the microenvironment (hypoxia, inflammation, necrosis, etc.), and also on the presence of different types of immune cells at the site of the transplanted cell localization.

### 4.3. Transplanted Apoptotic MSCs Clearance and the Mechanisms of Their Therapeutic Action

Phagocytosis is a key regulator of tissue homeostasis and cell turnover in adulthood and development. It is known that not only ApoBDs, but some apoptotic cells expressing appropriate signals, including find-me signals, eat-me signals, don’t-eat-me signals, and opsonins, do not break down into ApoBDs, but are efficiently ‘efferocyted’ by phagocytes [129,130]. This section addresses the mechanisms underlying the clearance of transplanted MSCs undergoing apoptosis in vivo, clearance of transplanted apoptotic MSCs, and clearance of transplanted MSC-derived ApoBDs with regard to their therapeutic activity.

In an elegant study, Luk et al. [131] showed that heat-inactivated MSCs that lost the capacity to respond to inflammatory stimuli and the ability to secrete trophic factors can still modulate the immune responses to sepsis, suggesting that MSCs can act as passive immunomodulatory vehicles. These inactivated MSCs did not suppress T cell proliferation and did not induce the formation of regulatory B cells, but modulated the function of monocytes in vitro. The authors concluded that the immunomodulatory effects of inactivated MSCs in vivo were associated precisely with their effect on the recipient’s monocytes [131]. A series of earlier studies conducted on animal models of the sepsis syndrome showed that apoptotic AD-MSCs exhibit protective effects associated with suppression of inflammation, inhibition of oxidative stress, and reduction of apoptosis in damaged organs [132,133,134,135]. Unfortunately, the papers described above there contain no data on the clearance of transplanted cells and the putative mechanisms underlying the therapeutic effects.

In addition to the rapid death of transplanted MSCs, it has also been proven that the apoptotic cells effectively undergo clearance in the recipient’s body. This process takes no more than a few hours, depending on the route of cell administration. Here the determination of cells able to rapidly phagocytize/efferocytize apoptotic MSCs in vivo is very important since in this case, phagocytic cells are a kind of mediator between infused MSCs, and the implementation of their therapeutic effects. Several studies have shown a pronounced immunosuppressive/immunomodulatory effect of transplanted apoptotic MSCs and MSC-ApoBDs in animal models of various diseases, including allergic airway inflammation [120,136], type 2 diabetes [113], liver fibrosis [123], acute liver injury, LPS-induced lung injury, and spinal cord injury [121], acute colitis [137]. In all the above works, apoptotic MSCs or MSC-ApoBDs were subject to efferocytosis mainly by macrophages.

Thus, in a type 2 diabetes (T2D) mouse model, i.v. infused human BM-MSC ApoBDs showed marked accumulation in the liver after 24 h, where they were mainly phagocytosed by Kupffer cells and monocyte-derived macrophages. Presumably, the “eat me” signal for efferocytosis, in this case, was calreticulin exposed on the ApoBD surface. Efferocytosis of MSC-ApoBDs resulted in transcriptional reprogramming of macrophages by proteins contained in the ApoBDs that have the potential to induce macrophage polarization into an anti-inflammatory M2 phenotype, including alpha-crystallin B chain (CRYAB), cAMP-dependent protein kinase type II-alpha regulatory subunit (PRKAR2A), receptor of activated protein C kinase 1 (RACK1) and vasodilator-stimulated phosphoprotein (VASP). Thus, efferocytosis of MSC-ApoBDs inhibited diet-induced obesity-induced macrophage activation in the liver, leading to an improvement in the chronic inflammatory environment in the T2D model [113].

Apoptotic MSCs pretreated with staurosporine exerted immunosuppressive effects in the lungs and inhibited allergic asthma to the same extent as live MSCs. When live CTV-labelled MSCs were intravenously administered to BALB/c mice, the CTV label was detected in both CD45^-^ stromal and CD45^+^ hematopoietic subpopulations in the lung. Tracking CTV^+^CD45^+^ cells over time showed that there is a hierarchy of phagocytic cell types that engulf CTV-labelled MSCs in the lungs. Ly6G^+^ neutrophils were the predominant cell type phagocytizing MSCs 10 min after i.v. injection, Ly6Chi, and Ly6Clo monocytes mainly engulfed transplanted MSCs after 1 h, CD11b^−^CD103^+^ type 1 conventional dendritic cells (cDC1)—after 2 h, and CD64+ interstitial macrophages—after 4 h. Interstitial macrophages and cDC1 were the main phagocytic cells at 8 h after i.v. injection of MSCs. During the first 8 h, uptake of CTV-labeled MSCs by CD11c^+^SiglecF^+^ alveolar macrophages remained constant at a low level, while uptake by CD11b^+^ cDC2 remained consistently low. Alveolar macrophages were shown to be a critical resident phagocytic population of the lungs, clearing both live and apoptotic MSCs, which underwent phenotypic changes after efferocytosis. Efferocytosis of apoptotic MSCs induced IFN-responsive genes and metabolic reprogramming of alveolar macrophages, followed by a marked inhibition of lung inflammation. Taken together, these data demonstrate that efferocytosis of apoptotic MSCs induces sustained changes in the immunometabolism and alveolar macrophage function that directly inhibit lung inflammation. The symptoms of the disease were suppressed even several weeks after the elimination of MSCs from the lungs [120].

The protective effect of the dead MSCs which undergo spontaneous cell death during in vitro culturing was also confirmed in four mouse models, including concanavalin A (ConA)- and carbon tetrachloride (CCl_4_)-induced acute liver injury, LPS-induced lung injury, and spinal cord injury. In the model of acute liver failure, i.v. transplantation of dead MSCs resulted in a significant decrease in activated NK cells and infiltrating neutrophils in the liver, while the number of Ly6Chi IL-10-producing macrophages was significantly increased. Dead MSCs contributed to the recruitment of macrophages to the liver by increasing PS and inducing a switch to an anti-inflammatory M2 phenotype [121].

After syngeneic transplantation of the CM-Dil-stained BM-MSCs into the fibrotic liver, CM-Dil^+^ signals were detected in CD11B^+^F4/80^+^ macrophages after 48 h, while dendritic cells and neutrophils showed minor signals. Thus, macrophage infiltration plays a major role in the clearance of infused apoptotic BM-MSCs. Moreover, the main part of the signal fell on Ly6Clo macrophages. Ly6Clo macrophages are highly restorative in fibrosis by upregulating various MMPs including MMP9, MMP12, and MMP13, which promote matrix degradation. The in vitro experiments have shown that MMP12 is the main effector in Ly6Clo macrophage-mediated resolution of fibrosis upon stimulation with BM-MSC ApoBDs. For the in vivo analysis, BM-MSCs, ApoBDs, and BM-MSCs pretreated with the caspase inhibitor Z-VAD-FMK were transplanted into fibrotic mice for the treatment of hepatic fibrosis. As expected, BM-MSC-infused livers showed low levels of hydroxyproline and α-SMA. However, treatment with only ApoBDs and BM-MSCs treated with Z-VAD-FMK showed no significant improvement in fibrosis compared to the PBS control group. These results demonstrate that the apoptosis of transplanted cells must occur in vivo in order to realize an anti-fibrotic therapeutic effect [123].

Endothelial cells (ECs) and platelets were also found among the cells capable of phagocytizing transplanted MSCs or MSC-ApoBDs and, therefore, acting as mediators between the infused cells and therapeutic effects. In a model of myocardial infarction, Liu et al. [109] found that MSCs transplanted intramyocardially into the border zone of the infarction release ApoBDs enhancing angiogenesis and improving functional recovery of the heart. Endothelial cells were identified as the main cells engulfing apoptotic MSCs in the myocardium. After intramyocardial injection, apoptotic MSCs were internalized by the PECAM1/CD31-positive ECs by 48 h. In addition, apoptotic MSCs were occasionally phagocytosed by VIM-positive cardiofibroblasts and TNNT2-positive cardiomyocytes in vivo. In vitro experiments confirmed that the main therapeutic effect of transplanted MSCs and/or apoptotic MSCs was associated with their phagocytosis by resident endothelial cells with subsequent regulation of macroautophagy/autophagy. In part, the improvement in the state of myocardial tissue could also be associated with the inhibition of the apoptosis of cardiomyocytes by apoptotic MSCs [109]. In another study [138] it was shown that ApoBDs derived from human deciduous pulp stem cells were also engulfed by ECs and increased the expression of angiogenic genes, leading to pulp revascularization and tissue regeneration in a nude mouse model of dental pulp regeneration. ApoBDs carried mitochondrial Tu translation elongation factor, which was thus transported to ECs and regulated angiogenic activation via the transcription factor EB-autophagy pathway. In an in situ beagle model of dental pulp regeneration, ApoBDs recruited endogenous ECs and facilitated the formation of dental pulp-like tissue rich in blood vessels [138].

hBM-MSCs ApoBDs corrected hemostasis in a hemophilia A model in factor VIII knockout mice through the upregulation of platelet activity following intraperitoneal injection. ApoBDs were already present on the surface of platelets 1 h after the injection. This interaction and subsequent platelet activation were mediated through the binding of Fas exposed on the surface of ApoBDs and FasL on the platelets. The ApoBD injection significantly enhanced the generation of platelet-derived microparticles. Although ApoBDs induced higher numbers of activated CD62P^+^ platelets and CD62P^+^ platelet-derived microparticles, they failed to elevate more TF^+^ platelets and TF^+^ platelet-derived microparticles. These data suggest that ApoBDs rebalance coagulation through upregulation of platelet activity without altering other hemostatic factors [112].

De Witte et al. [119] intravenously infused the PKH26-labeled human umbilical cord MSCs into the healthy BALB/c mice and found that the transplanted cells were phagocytosed by innate immune cells after 24 h. In the lungs, MSCs were engulfed mostly by the SSC^++^CD11b^++^ neutrophils and CX3CR^++^CD11b^++^ monocytes originating from the blood, and to a lesser extent by the CD68^+^CD11b^+^ macrophages. In the peripheral blood, the CX3CR^++^CD11b^++^ monocytes made up the main part of phagocytic cells, and neutrophils accounted for a very small part. In the liver, PKH26^+^ cells were predominantly engulfed by the CLEC4F^+^CD11b^+^ Kupffer cells, and a small percentage—by the CLEC4F^-^CD11b^++^ monocyte-derived macrophages and neutrophils [119]. In vitro experiments confirmed that human classical CD141^+^/CD16^-^ monocytes were able to phagocytize human umbilical cord MSCs, which led to their polarization towards the non-classical CD141^+^CD16^+^CD206^+^ phenotype and the expression of programmed death ligand-1 and IL-10. Monocytes primed with human UC-MSCs induced the formation of Foxp3^+^ regulatory T cells in mixed lymphocytic reactions. These results demonstrate that injected MSCs were rapidly ‘efferocytized’ by monocytes, which subsequently migrated from the lungs to other areas of the body. Phagocytosis of UC-MSCs induced phenotypic and functional changes in monocytes and subsequent modulation of the cells of the adaptive immune system [119]. Table 2 lists currently identified cell subpopulations capable of engulfing apoptotic MSCs or MSC-derived ApoBDs in vivo.

The following conclusions can be drawn from the above examples: (1) monocytes/macrophages are the main cells that efferocytosis apoptotic MSCs or MSC-derived ApoBDs upon systemic administration and subsequently play a decisive role in mediating, distributing, and transmitting the immunomodulatory effects of MSCs; (2) when administered locally, apoptotic MSCs can undergo phagocytosis not only by monocytes/macrophages but also by other types of resident cells, including, for example, endothelial cells or platelets, leading to their activation and stimulation of their functions. Thus, the main mechanism of action of apoptotic MSCs or MSC-derived ApoBDs is considered to be mainly their immunomodulatory effect, due to the presence of their obvious phagocytosis by immune cells after transplantation.

However, as shown in many studies, apoptotic cells and/or ApoBDs that circulate in the bloodstream, in addition to immunomodulation, are able to exert a wide variety of effects and perform various functions. For example, ApoBDs generated by tumor cells and entering the circulation have pleiotropic effects, including enhancement or suppression of antitumor immunity, promotion of metastasis, or increased procoagulant activity [140]. Many studies have also shown that apoptotic cells stimulate the proliferation of neighboring cells, triggering the so-called apoptosis-induced compensatory proliferation [141], as well as stem and progenitor cell proliferation, enhancing organ and/or tissue regeneration [142]. ApoBDs derived from cardiomyocytes and fibroblasts had different effects on myocardial regeneration in a model of doxorubicin-induced cardiomyopathy. Cardiomyocyte-derived ApoBDs stimulated the development of cardiomyocyte progenitor cells and myocardium regeneration, while fibroblast-derived ApoBDs stimulated endothelial progenitors and had no therapeutic effects [143]. The literature describes many examples of various effects, functions, and mechanisms of action of apoptotic cells and/or ApoBDs derived from various cell types [144]. Some of the mechanisms already described with regard to non-MSC-derived ApoBDs may also be mediated by apoptotic MSCs or MSC-derived ApoBDs. However, these potential mechanisms require further study.

## 5. Conclusions

The discovery of apoptotic cell death of MSCs after their transplantation undoubtedly became a powerful stimulus in the search for new potential mechanisms underlying the therapeutic action of these cells. However, this discovery also raised many questions that have to be answered. In particular, it remains unclear whether the process of apoptosis of transplanted MSCs is a prerequisite for their therapeutic effects. In this regard, it is also still necessary to establish if ApoBDs can be used for safe transplantation, and to what extent their effects are equivalent to those of live cells. Moreover, the differential separation of diverse EV subtypes, including the search for specific biomarkers, remains an unsolved problem at the moment.

## Figures and Tables

**Table 1:** The main characteristics of different types of EVs.

	Exosomes	Microvesicles	Apoptotic Bodies
Type of producing cells	All types of living cells	All types of living cells	Cells undergoing programmed cell death
Size	20–200 nm [33,34]	100–1000 nm [43]	50–5000 nm [57]
Biogenesis	Formed by inward budding of the late endosomal membrane, resulting in the formation of multivesicular bodies, which fuse with the plasma membrane and release exosomes into the extracellular space [31,32]	Formed by direct outward budding of the cell plasma membrane and subsequent pinching of the vesicles into the extracellular space [42]	Apoptosis initiation, plasma membrane blebbing and cell shrinkage, nuclear fragmentation, condensation and fragmentation of genetic material, and break-down of cellular contents into separate membrane-enclosed apoptotic bodies [58]
Protein markers	Tetraspanins (CD9, CD63, CD81), Annexin II and V, Flotillin-1 and -2, heat shock proteins 70 and 90, TSG101 [35], thrombospondin [106,107], syntenin-1 [112]	CD90, CD44, CD73 [46] CD133, CD271 [47] tetraspanins CD9, CD63 [49]	“find me” signals: CX3CL1 [78], PS-bound endogenous chemokines [81], tetraspanins [101], thrombospondin [91,95], Fas, Integrin alpha-5, Syntaxin-4, CD44, RhoA, Caveolin-1, Cavin1, Rab-5C, RPS25, Lamin B1, VDAC-2, Calnexin, and Calreticulin [112]
Membrane rearrangement	Yes Phosphatidylserine exposure [103,104,105]	Yes Phosphatidylserine exposure [44]	Yes Phosphatidylserine exposure [86]

**Table 2:** Identified cell subpopulations capable of engulfing apoptotic MSCs or MSC-derived ApoBDs in vivo.

Lung	Liver	Peripheral Blood/Bone Marrow	Cardiac Tissue/Dental Pulp
Ly6G^+^ neutrophils, Ly6Chi, and Ly6Clo macrophages, CD11b^−^CD103^+^ cDC1, CD64^+^ interstitial macrophages, CD11c^+^SiglecF^+^ alveolar macrophages, CD11b^+^ cDC2 [120]	Kupffer cells and monocyte-derived macrophages [113]	CX3CR^++^CD11b^++^ monocytes, neutrophils in peripheral blood [119]	PECAM1/CD31-positive ECs, VIM-positive cardiofibroblasts, TNNT2-positive cardiomyocytes [109,138]
SSC^++^CD11b^++^ neutrophils, CX3CR^++^CD11b^++^ monocytes, CD68^+^CD11b^+^ macrophages [119]	CD11B^+^F4/80^+^ macrophages, Ly6Clo macrophages [123]	CD105^+^CD73^+^CD44^+^ BM-MSCs and CD11b^+^ monocytes in bone marrow [139]	
	CLEC4F^+^CD11b^+^ Kupffer cells, CLEC4F^-^CD11b^++^ monocyte-derived macrophages, neutrophils [119]		

## Data Availability

Not applicable.
